# Editorial: Synthesis of novel photosensitizers for cancer theranostics

**DOI:** 10.3389/fchem.2023.1188243

**Published:** 2023-07-10

**Authors:** Jianhua Zou, Fuwu Zhang, Wenpei Fan, Ling Li, Zhen Yang

**Affiliations:** ^1^ Departments of Diagnostic Radiology, Surgery, Chemical and Biomolecular Engineering, and Biomedical Engineering, Yong Loo Lin School of Medicine, National University of Singapore, Singapore, Singapore; ^2^ Department of Chemistry, University of Miami, Miami, FL, United States; ^3^ State Key Laboratory of Natural Medicines and Jiangsu Key Laboratory of Drug Discovery for Metabolic Diseases, Center of Advanced Pharmaceuticals and Biomaterials, China Pharmaceutical University, Nanjing, China; ^4^ State Key Laboratory of Biotherapy and Cancer Center, Sichuan University, Chengdu, China; ^5^ Fujian Cross Strait Institute of Flexible Electronics (Future Technologies), Fujian Normal University, Fuzhou, China

**Keywords:** photosensitizer, theranostics, hypoxia, penetration depth, X-PDT

Cancer has posed a tremendous threat to the health of human beings worldwide, and an increasing number of people die of cancer every year (Siegel et al., 2022). Great efforts have been devoted to developing new therapeutic modalities for cancer treatment (Li et al., 2019a; Chen et al., 2019; Ma et al., 2019; Li L. et al., 2020; Wang et al., 2021; Li et al., 2023a). Phototherapy, including photodynamic and photothermal therapy, utilizes the photogeneration of reactive oxygen species (ROS) or heat to induce cell apoptosis (Zhen et al., 2017; Li et al., 2019b; Li L. et al., 2019; Yang and Chen, 2019; Li et al., 2020; Zheng et al., 2020; Zou et al., 2021a; Wu et al.). Solid tumors usually suffer from hypoxia which is strongly associated with tumor propagation, malignant progression, and resistance to therapy. However, several factors limit the widespread clinical use of photodynamic therapy (PDT), such as O_2_ shortage induced hypoxia and insufficient tissue penetration depth (Fan et al., 2016; Zhou et al., 2016; Liu et al., 2019; Qi et al., 2022). Therefore, new intelligent photosensitizers should be designed and synthesized to achieve better phototherapeutic efficacy. Apart from cancer therapy, PDT has been universally utilized in a variety of fields, such as plastic and re-constructive surgery. Wu et al. from Shanghai Jiaotong University has summarized the application of PDT in benign pigmented lesion, vascular malformation, inflammatory lesion, etc.

In recent years, great efforts have been devoted to relieving hypoxia, for example, *in situ* oxygen generation or delivering oxygen to the tumor (Yang et al., 2017; Lin et al., 2018; Shen et al., 2022; Li et al., 2023b; Chen et al., 2023). Representative work by Jianlin Shi is the *in situ* generation of oxygen, typically transition metal oxides, such as MnO_2_ in the TME. The degradation of MnO_2_ not only releases oxygen but also leads to the metabolism of Mn^2+^ (Fan et al., 2015). Another way is to deliver molecular oxygen to the tumor region, typical of which is the utilization of Food and Drug Agency (FDA) approved perfluorocarbon capable of carrying the oxygen (Cheng et al., 2015; Wang W. et al., 2019). Perfluorocarbon proves to be a safe drug with excellent bio-compatibility. In addition, the fractionated delivery of singlet oxygen by chemical storage is an efficient approach to treatment of hypoxic tumors. In the process, singlet oxygen is usually captured by the moiety, such as pyridione, anthracene with laser irradiation (Zou et al., 2020; Zou et al., 2021b). Then it will be released when the laser is off. Fractionated delivery of singlet is a kind of mild PDT and diminishes the damage of blood vessels, thus contributes to supplying oxygen during blood circulation. Apart from relieving hypoxia, diminishing oxygen consumption with oxygen-independent therapy is considered as another effective way, for example, type I PDT (Ding et al., 2011; Wang Y. et al., 2019; Zhuang et al., 2020). Different from type II PDT, type I PDT is based on the sensitization of photosensitizers to generate superoxide/hydroxyl radicals which may derive from not only molecular oxygen, but also water or hydrogen peroxide. A classic example is the radiosensitization of TiO_2_ by X-ray leads to the efficient generation of hydroxyl radicals (Zhang et al., 2014). In recent years, photosensitizers with NIR absorbance may also act similarly. In the Research Topic, Cui et al. from Xiangyang Central Hospital synthesized a semiconducting polymer (PDPP) and encapsulated it with hydrophilic PEG-PDPA to enhance the electron transfer for type I PDT. PDPP NPs show high superoxide radical generation ability. Both *in vitro* and *in vivo* study demonstrate PDPP NPs with considerably high phototoxicity against human cervical cancer. Apart from hydrophilic PEG, extracellular vesicles (EVs) can also be used as the platform for the delivery of photosensitizers (Tong et al.). Tong et al. from Shandong First Medical University have systematically summarized the passive and active loading strategies of photosensitizers into EVs, the advantages and disadvantages of EV based delivery nanoplatform. According to their statistical analysis, cancer cells (23.6%), stem cells (22.9%), and HEK293 (21.7%) derived EVs were most commonly used in preclinical studies. This may be because researchers are trying to take advantage of the homing and immune escaping properties of EV pararenal cells, such as cancer cells and stem cells (Escude Martinez de Castilla et al., 2021).

Activatable nano-platform for cancer therapy is attracting broad interest (Turan et al., 2016; Hu et al., 2018; Zou D. et al., 2021). Glutathione (GSH) with reductivity exists universally in cancer cells. Designing nanomaterials for depletion of GSH may enhance the therapeutic efficacy of PDT. Tang et al. from Guangdong Medical University prepared a smart nanoplatform for enhanced photo-ferrotherapy against hepatocellular carcinoma. Given that the overexpression of hydrogen sulfide (H_2_S) in colorectal cancer (CRC), Li et al. from the National Institutes of Health (NIH) developed a novel metal-organic framework (MOF) composed of meso-Tetra (4-carboxyphenyl) porphine (TCPP) and ferric ion (Fe^3+^) through a facile one-pot process. The MOF is capable of depredating in response to the high content of H_2_S in tumor microenvironment of CRC.

NIR-II fluorescence imaging benefits from deeper penetration, less tissue scattering and diminished auto-fluorescence (Hong et al., 2017; Tian et al., 2019; Pei et al., 2021). Niu et al. from the First Affiliated Hospital of Fujian Medical University reported a biomineralized hybrid nanodots (Cu_x_Mn_y_S_z_@BSA@ICG, ICG = indocyanine green) for tumor therapy via NIR-II fluorescence for photothermal therapy. Cu_x_Mn_y_S_z_@BSA@ICG converts endogenous hydrogen peroxide (H_2_O_2_) into highly active hydroxyl radical (•OH) via Fenton reaction, and effectively produces reactive oxygen species (ROS) after being exposed to 808 nm laser irradiation. This results in eliciting a ROS storm, leading to the regression of tumor.

This Research Topic has attracted extensive interest from researchers who would like to seek new therapeutic methods for better understanding the relationship between the structure and therapeutic efficacy. The knowledge generated here not only benefits the researchers focused on synthetic chemistry and biomaterials but also adds to the understanding of cancer treatment for pre-clinical application. Further investigation should still be continued for cancer phototheranostics.
